# Anti-TGF-β Antibody, 1D11, Ameliorates Glomerular Fibrosis in Mouse Models after the Onset of Proteinuria

**DOI:** 10.1371/journal.pone.0155534

**Published:** 2016-05-17

**Authors:** Xiaoyan Liang, H. William Schnaper, Taiji Matsusaka, Ira Pastan, Steve Ledbetter, Tomoko Hayashida

**Affiliations:** 1 Department of Pediatrics, Feinberg School of Medicine, Northwestern University, Chicago, Illinois, United States of America; 2 Department of Internal Medicine, Tokai University School of Medicine, Isehara, Kanagawa, Japan; 3 Laboratory of Molecular Biology, Center for Cancer Research, National Cancer Institute, Bethesda, Maryland, United States of America; 4 Sanofi-Genzyme R&D Center, Framingham, Massachusetts, United States of America; Fondazione IRCCS Ospedale Maggiore Policlinico & Fondazione D’Amico per la Ricerca sulle Malattie Renali, ITALY

## Abstract

Fibrosis is a final common pathway leading to loss of kidney function, in which the fibrogenic cytokine, transforming growth factor β (TGF-β), plays a central role. While previous studies showed that TGF-β antagonism by various means prevents fibrosis in mouse models, clinical approaches based on these findings remain elusive. 1D11 is a neutralizing antibody to all three isoforms of TGF-β. In both adriamycin (ADR)-induced nephropathy and NEP25 podocyte ablation nephropathy, thrice-weekly intraperitoneal administration of 1D11 from the day of disease induction until the mice were sacrificed (day 14 for ADR and day 28 for NEP25), significantly reduced glomerular COL1A2 mRNA accumulation and histological changes. Consistent with our previous findings, proteinuria remained overt in the mice treated with 1D11, suggesting distinct mechanisms for proteinuria and fibrogenesis. Podocyte numbers determined by WT1 staining were significantly reduced in NEP25-model glomeruli as expected, while WT1-positive cells were preserved in mice receiving 1D11. Even when 1D11 was administered after the onset of proteinuria on day 3, 1D11 preserved WT1-positive cell numbers in glomeruli and significantly reduced glomerular scar score (2.5 ± 0.2 [control IgG] vs. 1.8 ± 0.2 [1D11], *P* < 0.05) and glomerular COL1A2 mRNA expression (19.3 ± 4.4 [control IgG] vs. 8.4 ± 2.4 [1D11] fold increase over the healthy control, *P* < 0.05). Transmission electron microscopy revealed loss of podocytes and denuded glomerular basement membrane in NEP25 mice with disease, whereas podocytes remained attached to the basement membrane, though effaced and swollen, in those receiving 1D11 from day 3. Together, these data suggest that TGF-β neutralization by 1D11 prevents glomerular fibrosis even when started after the onset of proteinuria. While overt proteinuria and podocyte effacement persist, 1D11 prevents total podocytes detachment, which might be a key event activating fibrogenic events in glomeruli.

## Introduction

Fibrosis is a final common event in many glomerular disorders, leading to loss of kidney function. Among multiple factors that are involved in fibrogenesis, transforming growth factor β (TGF-β) has been implicated as a major fibrogenic cytokine both in vivo and in vitro (reviewed in [1]). Overexpression of TGF-β in mice is sufficient to induce proteinuria and subsequent glomerular sclerosis [2–4], and many genes encoding extracellular matrix, such as collagen and fibronectin, are TGF-β responsive [5, 6]. Furthermore, urinary TGF-β excretion is increased in patients with nephrotic syndrome [7], IgA nephropathy [8] and focal segmental glomerulosclerosis (FSGS) [9]; and urinary TGF-β levels correlate with ECM accumulation in FSGS [10, 11] and may be a predictive marker for disease progression [12, 13]. Together, these reports indicate a causal link between TGF-β expression levels and kidney fibrosis, supporting our intent to target TGF-β in preventing fibrosis. At least in rodent models, several means to interfere with TGF-β action such as administration of natural TGF-β antagonist, decorin [14, 15]; siRNA-mediated gene silencing of the TGF-β signaling molecule, Smad [16], or overexpression of inhibitory Smad, Smad7 [17]; and administration of anti-TGF-β antibodies in diabetic animals [18, 19] have proven to be effective in preventing kidney fibrosis.

We recently reported that, in Adriamycin (ADR)-induced nephropathy, intraperitoneal administration of soluble extracellular domain of type II TGF-β receptor fused with Fc portion of IgG (sTβ RII-Fc) prevented kidney fibrosis, while proteinuria persists at least within the 2-week duration of the experimental time frame [20]. sTβ RII-Fc was also shown to prevent fibrotic changes in the Thy1 rat[21, 22]. On the other hand, specific inhibition of a γ isoform of phophoinositide 3-kinase (PI3K) prevented podocyte loss and proteinuria, as well as fibrotic changes [20]. These finding indicate that mechanisms that mediate initial podocyte damage and subsequent fibrogenesis are distinct, raising the possibility that TGF-β inhibition could halt progression of fibrosis even after the onset of proteinuria.

1D11 is a murine monoclonal antibody that neutralizes all three mammalian isoforms of TGF-β [23]. Administration of 1D11 has been reported to prevent progression of kidney fibrosis in several rodent models of kidney diseases, including streptozotocin (STZ)-induced diabetic rats [24, 25], Thy1 nephritis in rats [26], 5/6 nephrectomy uremic rats [27] and Dahl salt-sensitive hypertensive rats [28]. 1D11 also prevents tubular damage in the unilateral ureteral obstruction model [29] or cyclosporine-induced nephropathy [30], further indicating that TGF-β inhibition could prevent fibrosis regardless of the initial cause of injury.

Here, we tested the efficacy of 1D11 administered after the onset of proteinuria, using the ADR and NEP25 podocyte ablation model. Our results indicate that TGF-β inhibition can ameliorate progressive fibrosis even after the initiating insult in these models, and suggest that multiple mechanisms of podocyte damage and proteinuria may lead to a common, TGF-β -mediated mechanism of glomerulosclerosis.

## Materials and Methods

### Ethics Statement

The present study was conducted in strict accordance with the recommendations in the Guide for the Care and Use of Laboratory Animals of the National Institutes of Health. All protocols were approved by the Institutional Animal Care and Use Committee of the Northwestern University (protocol number 2013–1880). When animals were sacrificed, mice were given Ketamine (50mg/kg BW) / Xylazine (5mg/kg BW) mixture intraperitoneally and loss of sensation was verified prior to any invasive maneuver is applied.

### Animal models

#### Adriamycin nephropathy

Male 129x1/Svj mice (8 weeks old, stock #000691) were purchased from Jackson Laboratory (Bar Harbor, MN). A single dose of adriamycin (15 mg/kg body weight; BW) was intravenously injected from tail vain to induce nephropathy as previously described [20].

#### NEP25 podocyte ablation model

NEP25 mice that aberrantly express human CD25 in podocytes under the control of the mouse nephrin promoter [31] were generated at the Matsusaka Laboratory and a local colony was maintained at the Center for Comparative Medicine, Northwestern University. A chimeric toxin, LMB2, that is comprised of a mutant form of *Pseudomonas* exotoxin (PE38) fused with the Fv portion of anti-Tac (human CD25) antibody [32], was generated at the Pastan laboratory and stocked in -80°C after titer determination.

#### Anti-TGF-β antibody preparation and administration

1D11 is a murine IgG1 monoclonal antibody that neutralizes all three TGF-β isoforms. The 1D11 antibody and its isotype-matched murine IgG1 control (13C4) were produced by Genzyme Corporation. Stock solutions (10 mg/ml) were stored as small aliquots at -80°C and thawed on the day of injection. In the pretreatment experiments, mice received either 1D11 or 13C4 intraperitoneally one day before and on the day of disease induction, followed by every-other-day intraperitoneal administration until sacrifice. In the post-induction treatment experiments, the presence of proteinuria was confirmed with a spot urine sample 3 days after LMB2 administration and those mice that had proteinuria (100 μg/ml or more) were randomly allocated to either 1D11 or 13C4 group.

#### Sample collection

The day before sacrifice, the mice were kept overnight in metabolic cages to collect urine samples. Thereafter, under deep anesthesia with Ketamine/Xylazine, a blood sample was collected by direct cardiac puncture and the kidneys halves were harvested and either preserved in 10% natural buffered formalin for histological analyses, frozen in OCT compound for cryosectioning, or snap frozen for RNA and protein evaluation. In selected experiments, portions of kidney were infused with 2.5% glutaraldehyde solution for sample processing for transmission electron microscopic (TEM) evaluation.

### Histological evaluation

Formalin-fixed kidney samples were embedded in paraffin and cut in 4-μm sections, followed by Periodic Acid Schiff (PAS) or silver staining by the Mouse Histology and Phenotyping Laboratory of Northwestern University. Stained sections were scanned by the TissueFAXS system (TissueGnostics, Vienna, Austria) as composite images of 200x resolution and approximately 100 glomeruli in each section were manually depicted as regions of interest (ROIs). Glomerular scar score was determined for each ROI by the following scoring scale; “0” = no scar, “1” = < 25%, “2” = 25–50%, “3” = 50–75% and “4” = 75–100% of the glomerular surface is occupied by fibrotic changes. Individual images were acquired with a Zeiss Axioscope equipped with a CRI Nuance spectral camera. Samples for transmission electron microscope (TEM) evaluation were processed by standard methods [33] and 70 nm-thick sections of EPON-embedded sample were evaluated by FEI Tecnal Sprit G2 TEM (120 kV, FEI, Hilsboro, OR). All of the imaging work was performed with instruments managed by the Center for Advanced Microscopy, Northwestern University.

### Immunohistochemistory

Kidney samples freshly sectioned from formalin-fixed, paraffin-embedded blocks were subjected to innumohistochemical detection of WT1 (SC-192, rabbit polyclonal 1:50; Santa Cruz Biotechnology, Santa Cruz, CA), pSmad3 (ab51177, rabbit monoclonal, 1:50; Abcam, Cambridge, MA) or type I collagen (20R-CR025, rabbit polyclonal, 1:500; Fitzgerald) with a standard protocol [20]. Briefly, after deparaffinization and rehydration, antigen was exposed by steaming with Target Retrieval Solution (DAKO/Agilent Technologies, Carpinteria, CA) and endogenous peroxidase and biotin activities were quenched with 3% H_2_O_2_ in methanol or biotin blocking solution (Life technology/Invitrogen), respectively. After application of a primary antibody overnight at 4°C, the bound antibody was amplified with goat anti-rabbit IgG (H+L) conjugated with biotin (ab3720, 1:200; Abcam) and avidin-HRP (#18–4100, eBioscience, San Diego, CA), followed by DAB reaction (Life Technology/Invivrogen) and hematoxylin counter staining. Positive staining within manually defined glomeruli (ROI) was analyzed using HistoQuest software (TissueGnositics) with the pixel characteristics defined for WT1 or pSmad3-positive (brown) and negative (blue) nuclei.

### Urine and serum sample analyses

Albumin and creatinine concentrations in the urine and serum, and urea concentration in the serum samples were determined using ELISA kits according to the manufacturer’s directions (Exocell, Philadelphia, PA).

### mRNA expression analyses

Glomeruli were captured from OCT-embedded, cryosectioned kidney samples using PALM laser-capturing microdissection system (Carl Zeiss microImaging GmbH, Munich, Germany) as previously described [34, 35]. Total RNA was extracted with RNeasy kit (Quagen, Valencia, CA) as instructed by the manufacturer, and then reverse-transcribed with the iScript cDNA synthesis kit (Bio-Rad Laboratories) for quantitative PCR using the SsoAdvanced Universal Supermix (Bio-Rad Laboratories) with the CFX96 Touch real-time PCR detection system (Bio-Rad). Real-time data were collected for 40 cycles of 95°C, 10 s, 57°C, 45 s, and 75°C, 30 s. Samples were run in duplicates and relative expression of the gene of interest was estimated by the ∆∆Ct method using 18S as a reference gene. Primers used were custom-synthesized by Integrated DNA Technology (Coralville, IA) and detailed primer information is provided in Table 1.

**Table 1:** Primer sequences used in this study.

Name	Species	Accession #	Forward 5’ > 3’	Reverse 5’ > 3’
COL1A2	mouse	NM_007743	CTGGAACAAATGGGCTCACTG	CAGGCTCACCAACAAGTCCTC
Fibronectin	mouse	NM_001276413	GCAGTGACCACCATTCCTG	CCTGTCTTCTCTTTCGGGTTCA
TGF-β1	mouse	NM_011577	CCGCAACAACGCCATCTATG	CTCTGCACGGGACAGCAAT
TGF-β2	mouse	NM_009367	GGCTTTCATTTGGCTTGAGATG	CTTCGGGTGAGACCACAAATAG
TGF-β3	mouse	NM_009368	CGCTACATAGGTGGCAAGAA	CAAGTTGGACTCTCTCCTCAAC
ribosomal18S	mouse	NM_011296	AGTTCCAGCACATTTTGCGAG	TCATCCTCCGTGAGTTCTCCA

### Statistical analyses

Statistical analyses were performed using GraphPad Prism version 4.0 for Macintosh (GraphPad Software, San Diego, CA) for t-test or one-way analysis of variance followed by post-hoc analysis. *P* < 0.05 was considered significant.

## Results

**Fig 1:**
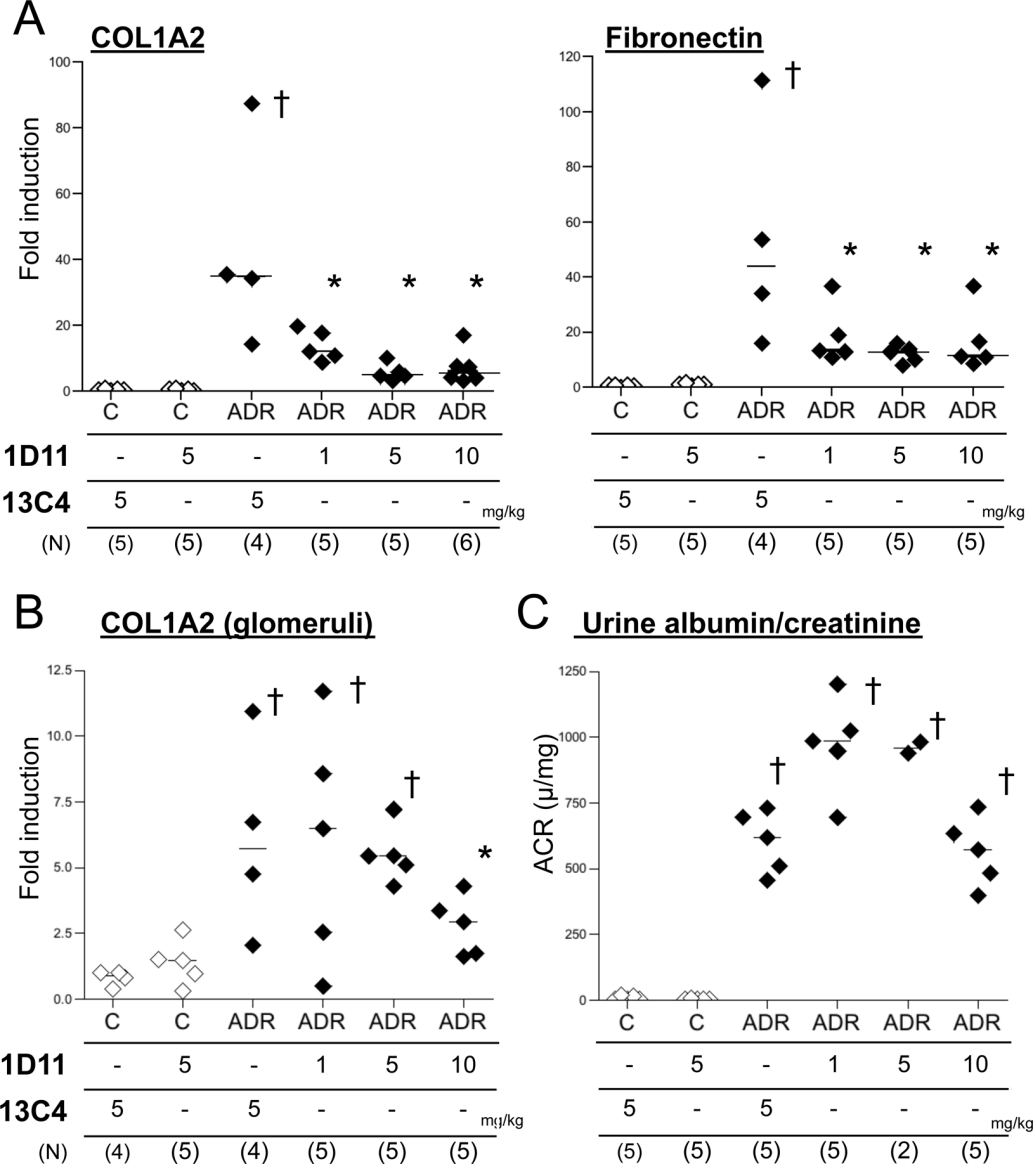
1D11 prevents fibrosis in ADR nephropathy. (A) RNA extracted from a portion of the whole kidney was analyzed for mRNA by qPCR. † *P* < 0.01, compared to healthy control, * *P* < 0.01 compared to disease control (3^rd^ column from the left), by Bonferroni’s post-hoc test following one-way ANOVA. (B) Glomerular mRNA content was analyzed with laser-captured glomeruli from cryosectioned kidney samples. † *P* < 0.01, compared to healthy control, * *P* < 0.01 compared to disease control, by post-hoc test with Welch correction following Kruskal-Walis one-way ANOVA. (C) Urine albumin and creatinine concentrations were analyzed by ELISA. † *P* < 0.01, compared to healthy control by Boferroni’s post-hoc test following one-way ANOVA.

**Fig 2:**
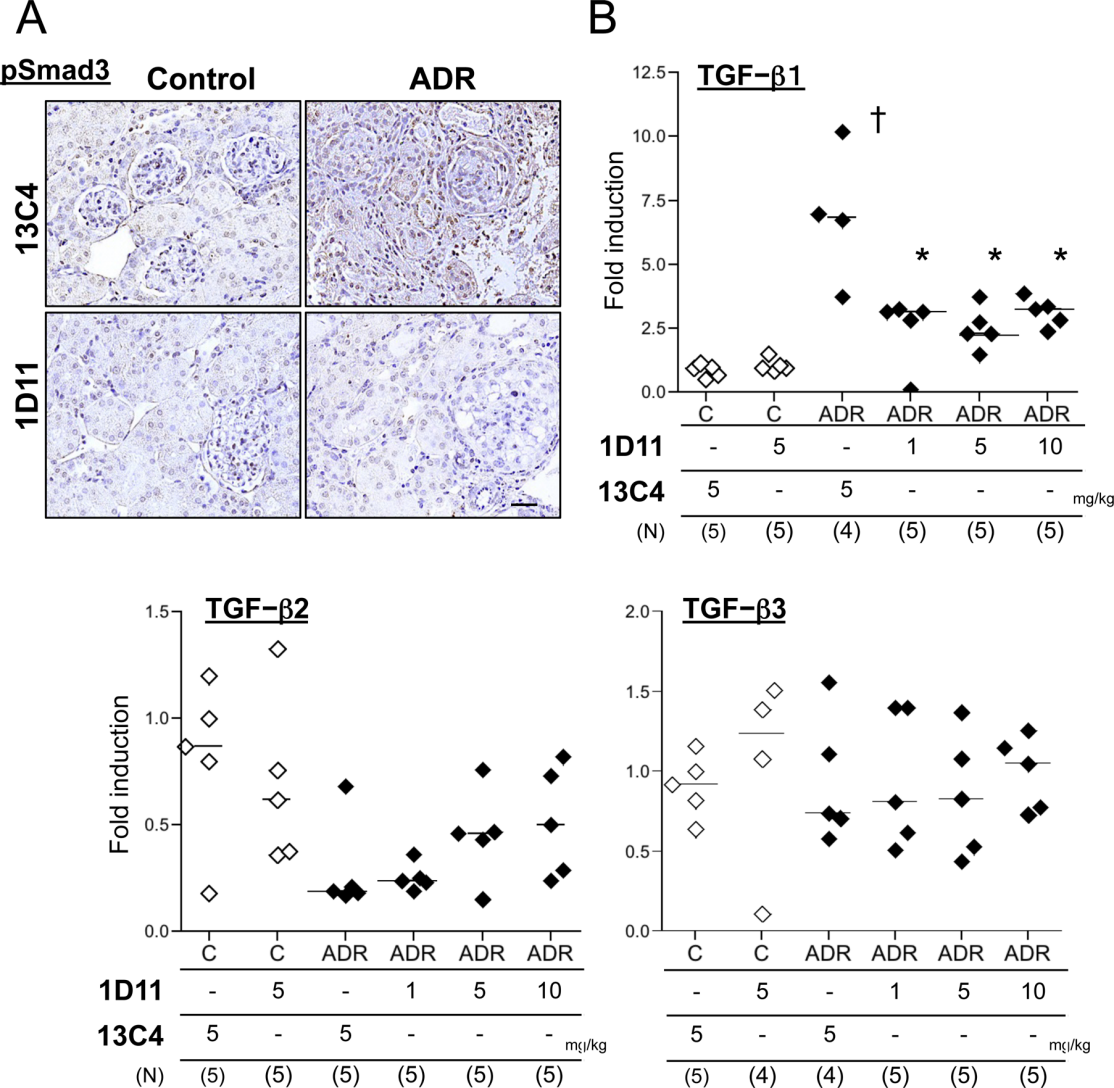
1D11 suppresses TGF-β signaling *in vitro*. (A) Formalin-fixed, paraffin-embedded kidney sections were stained for pSmad3 (brown nuclei) followed by counterstaining with hematoxylin (blue nuclei). Scale bar = 100 μm. 40x objective with oil. (B) mRNA expression of TGF-β isoforms was evaluated with whole kidney lysates. † *P* < 0.01, compared to healthy control, * *P* < 0.01 compared to disease control (3^rd^ column from the left), † *P* < 0.01, compared to healthy control by Bonferroni’s post-hoc test following one-way ANOVA.

Previously, we reported that administration of sTβRII-Fc, which sequesters ambient TGF-β, prevents fibrotic changes in ADR nephropathy in mice [20]. Here, we first tested whether 1D11, an antibody to all 3 isoforms of TGF-β, similarly prevents fibrosis in the ADR nephropathy model. 1D11 was well tolerated by the mice, except that some mice receiving 10 mg/kg showed moderate signs of distress (weight loss and decreased activity) at the end of the experiment. Consistent with our previous findings, 1D11 significantly decreased expression of COL1A2 and fibronectin mRNA in whole kidney (Fig 1A). Glomerular COL1A2 expression was also reduced but a higher dose of 1D11 was required (Fig 1B). On the other hand, levels of proteinuria were not significantly different comparing 1D11 with control 13C4 treated mice (Fig 1C). Nuclear pSmad3 staining indicated activation of TGF-β signals in mouse kidneys after ADR administration, which was abrogated in the mice treated with 1D11 (Fig 2A). TGF-β1 mRNA expression in ADR-treated mouse kidney was also elevated (Fig 2B), but was suppressed by 1D11 as has been previously reported [23]. 1D11 also decreases TGF-β2 and 3 expressions [23]. However, ADR administration did not significantly stimulate expression of TGF-β2 and 3 (Fig 2B), indicating that the preventive effect of 1D11 is mainly via suppression of TGF-β1 signals.

**Fig 3:**
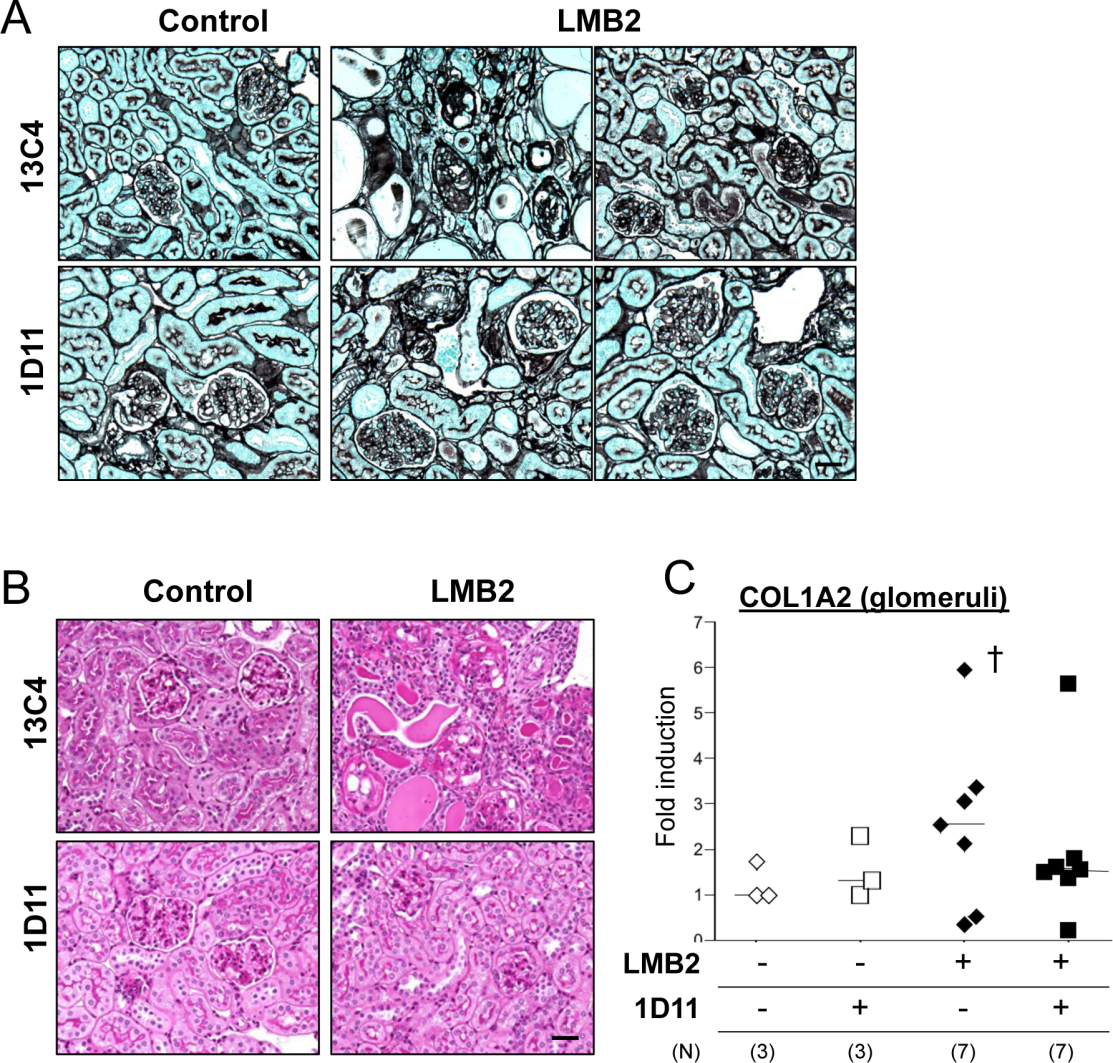
1D11 prevents fibrosis in the NEP25 podocyte ablation model. (A and B) Representative images of NEP25 mouse kidney samples with silver- (A) and PAS staining (B). Disease was induced by injection of LMB2, and 1D11 or 13C4 (control IgG) was administered on day -1 and day 0, followed by further injections every other day until sacrifice at day 28. Scale bar = 100 μm. 40x objective. (C) Glomerular mRNA content was analyzed with laser-captured glomeruli from cryosectioned kidney samples. † *P* < 0.05 compared to healthy controls by t-test with Welch correction.

**Fig 4:**
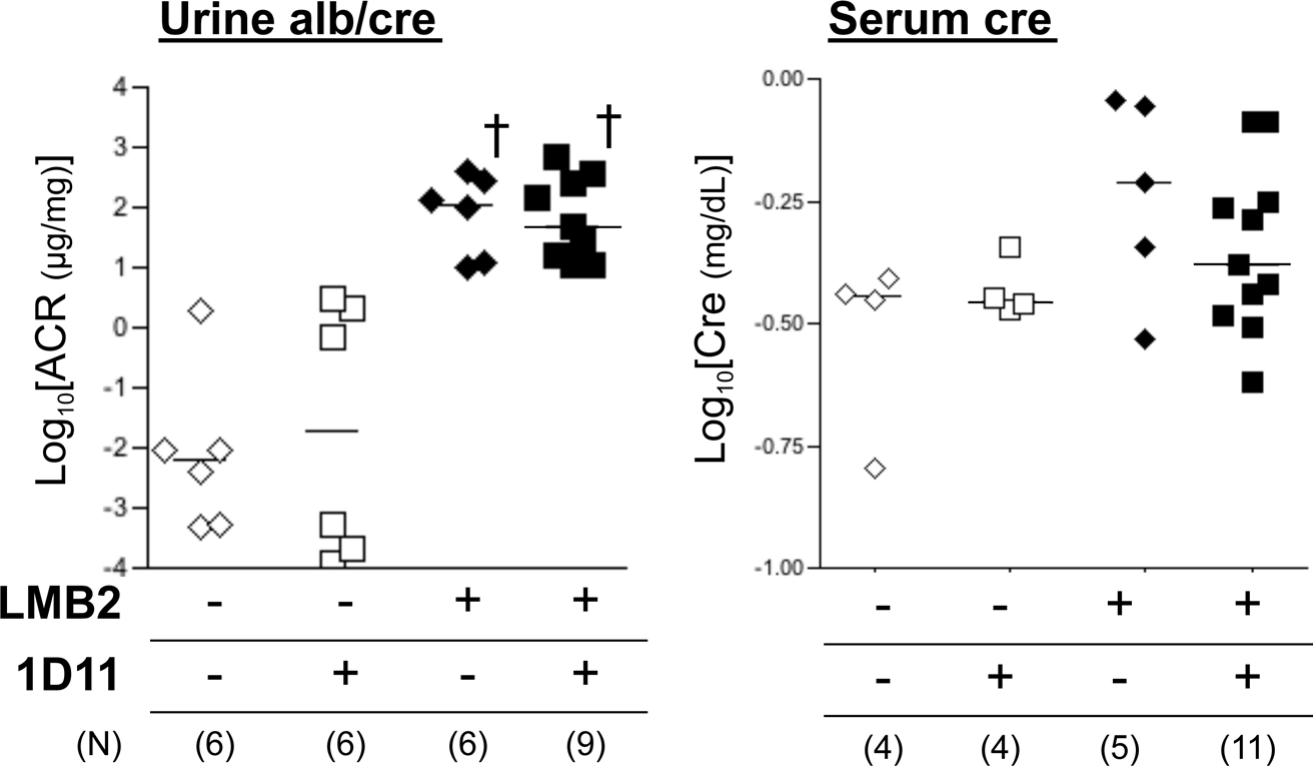
1D11 does not affect proteinuria in NEP25 model. Urine albumin, and urine and serum creatinine concentations were analyzed by ELISA kits. Urine albumin to creatinine ratio (left) and serum creatinine concentration (right) were shown after logarithmic transformation. † *P* < 0.05 compared to healthy controls by Bonferroni’s post-hoc test compared to no disease controls, following one-way ANOVA.

**Fig 5:**
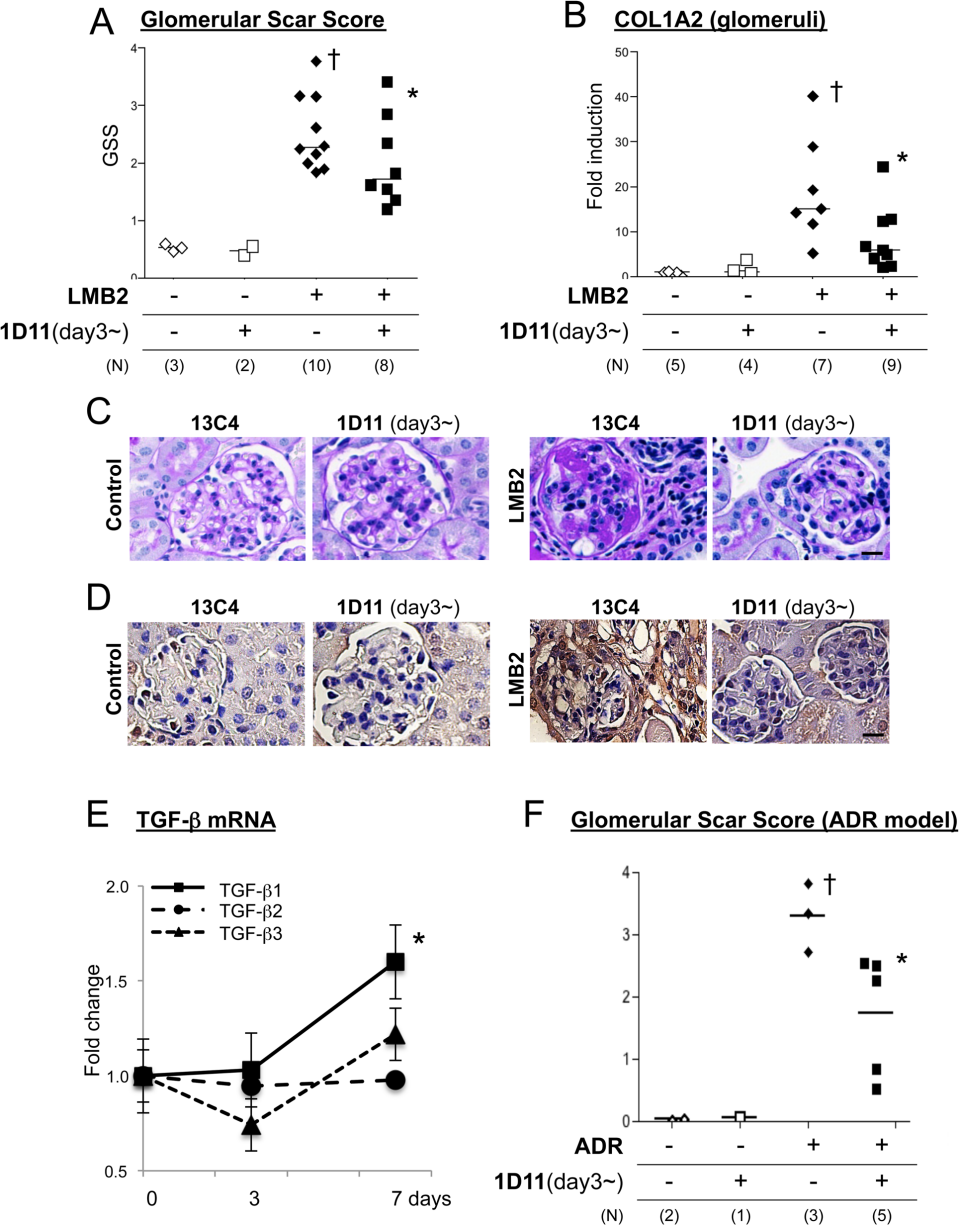
1D11 ameliorates fibrosis when administered after the onset of proteinuria prevents glomerular fibrosis. (A) Three days after disease induction, spot urine was tested for proteinuria and mice that were positive of proteinuria were randomly allocated to either 1D11 or 13C4 group and histology was evaluated day 28. Each dot represents the median of the glomerular scar score, which was evaluated by a blinded observer in approximately 80–100 glomeruli of a PAS-stained section. † *P* < 0.05 compared to healthy controls by Dunn’s post-hoc test compared to no disease controls, following Kruskal-Walis one-way ANOVA. * *P* < 0.05 compared to LMB2 only group by Mann Whitney test. (B) Glomerular mRNA content was measured with laser-captured glomeruli from cryosectioned kidney samples. † *P* < 0.05 compared to healthy controls by Bonferroni’s post-hoc test compared to no disease controls, following one-way ANOVA. * *P* < 0.05 compared to LMB2 only group by unpaired, two-tailed, *t*-test. (C and D) Representative PAS staining images (C) and immunohistochemical evaluation of type I collagen (D) from the experiment analyzed in A and B are shown. Scale bar = 20 μm. 20x objective. (E) mRNA expression of three TGF-β isoforms in whole kidney of NEP25 mice was analyzed by qPCR. Relative changes compared to day 0 are shown. *P < 0.05 compared to day 0. (F) Mice were injected with 1.0 mg/kg BW of adriamycin via tail vain, and 1D11 administration was started at day 3, followed by every other day, and mice were sacrificed at day 10 for evaluation. Glomerular scar score was evaluated as described for Fig 5A. † *P* < 0.05 compared to healthy controls. * *P* < 0.05 compared to LMB2 only group by Mann Whitney test.

While ADR nephropathy demonstrates histological changes resembling human glomerular disorders, the pathogenesis of this lesion may also involve endothelial cell damage [36, 37] and/or inflammation [38], which may precede podocyte damage that in general is a primary event in majority of human disorder [39]. To test the anti-fibrotic efficacy of the TGF-β antibody in a more specific podocyte-damage model, we next utilized the NEP25 mouse. This mouse expresses human CD25 (hCD25) specifically in podocytes under the control of the mouse nephrin promoter, and administration of a chimeric toxin, LMB2 [32], that is composed with a Fv portion of an anti-hCD25 (Tac) antibody and a mutant form of pseudomonas exotoxin (PE38), ablates hCD25-expressing podocytes, resulting in proteinuria and subsequent fibrosis in a dose of LMB2-dependent manner [31]. In our pilot experiments, 0.65 to 1.0 ng/kg BW LMB2 caused moderate proteinuria but minimal fibrotic changes (not shown), while 2.5 ng/kg BW LMB2 induced significant proteinuria by day 3 and global fibrosis was established by day 28 (Fig 3A and 3B). Based on the data obtained from experiments with the ADR nephropathy model and the length of treatment in the NEP25 model, we chose the dose of 1D11 anti-TGF-β antibody or 13C4 control IgG as 3 mg/kg BW. NEP25 mice receiving 1D11 demonstrated less glomerular fibrosis as shown by representative silver staining images in Fig 3A, and glomerular COL1A2 mRNA expression was not significantly different from healthy controls (Fig 3C). On the other hand, 1D11 did not affect the degree of proteinuria induced by LMB2 (Fig 4A). Serum creatinine levels were only moderately elevated in NEP25 mice, and were not significantly different between the 1D11 and 13C4 groups (Fig 4B). These results are consistent with our previous findings in the ADR nephropathy model, in that TGF-β antagonism prevents progression of glomerular fibrogenesis despite persistent proteinuria, suggesting that TGF-β mediates mainly fibrogenesis, the mechanism for which is distinct from that of proteinuria. If so, administering 1D11 after the onset of podocyte damage might also be effective in halting the progression of fibrosis. To test this possibility, mice were evaluated for the presence of proteinuria three days after the LMB2 administration, and those that were proteinuria positive were randomly allocated to either 1D11 or 13C4 groups and evaluated 4 weeks later. As shown in Fig 5A, 5B and 5C, glomerular scar score, as well as glomerular COL1A2 mRNA expression, were significantly lower in the mice that received 1D11 compared to those receiving control IgG. Delayed administration of 1D11 also significantly reduced extensive Type I collagen accumulation observed in glomeruli of mice received LMB2 (Fig 5D). TGF-β1 mRNA was minimally increased at day3, and significantly rose by day 7 (Fig 5E), supporting our timing for our delayed treatment. Delayed administration of 1D11 protected from glomerular sclerosis in ADR mice (Fig 5F).

**Fig 6:**
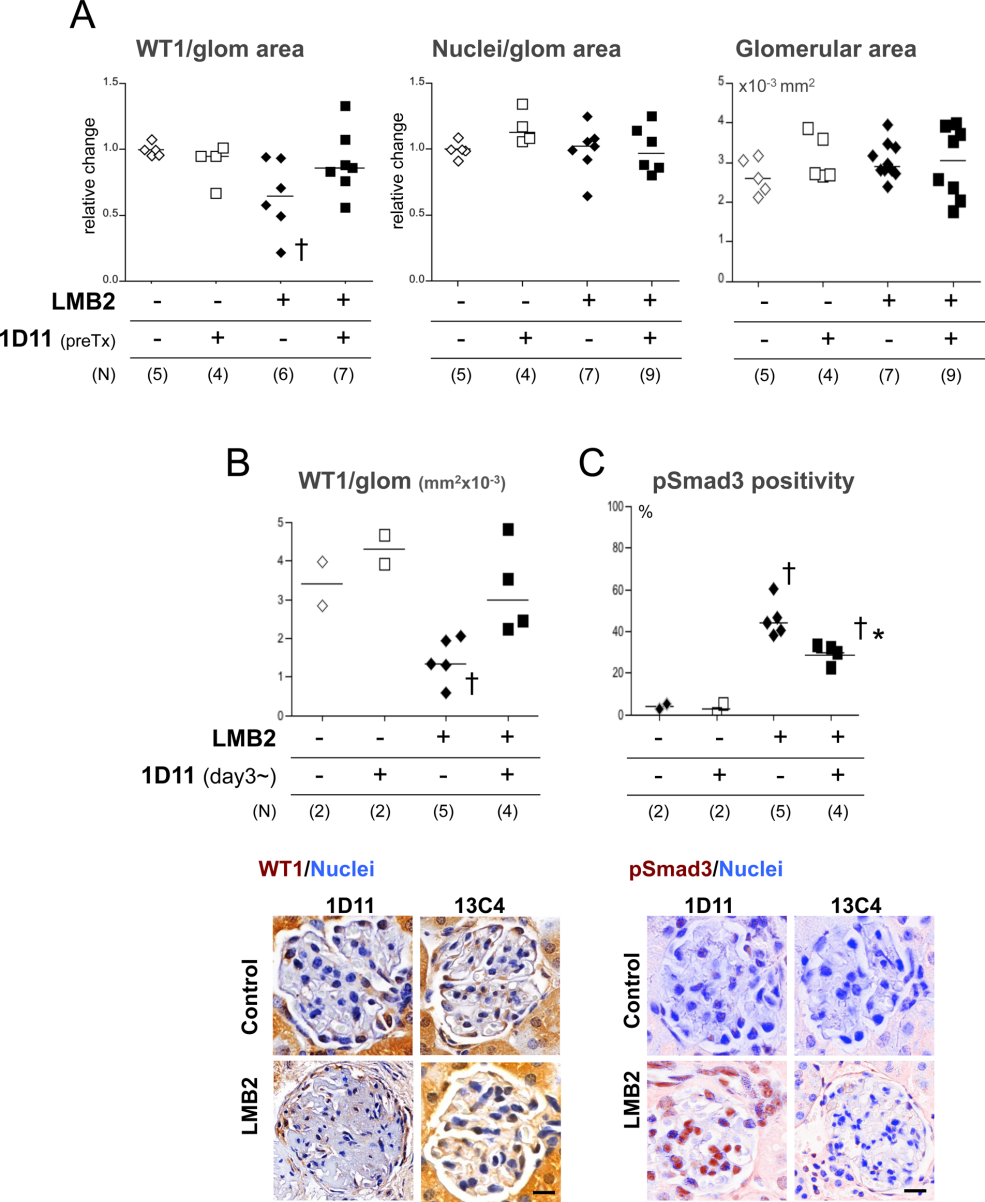
1D11 prevents podocyte loss (A) WT-1 positive cells numbers per 10^−3^ mm^2^ were counted in ~100 glomeruli in a PAS-stained kidney section using the TissueGnostics system. Each dot represents the median of WT-1 positivity in a mouse, corrected for a healthy control as 1 from 3 independent experiments. (B) WT-1 positivity was also analyzed with mice that received 1D11 after the onset of proteinuria positive of proteinuria or relevant control mice. Each dot represents median of WT-1-positive cell numbers per 10^−3^ mm^2^ in glomeruli. † *P* < 0.05 compared to healthy controls by Dunn’s post-hoc test compared to no disease controls, following Kruskal-Walis one-way ANOVA. Representative images of glomeruli are shown in the bottom panel. WT1: DAB, Nuclei: Hematoxylin. Scale bar = 20 μm. 40x objective. (C) Percentage of pSmad3 positive nuclei over total nucleus numbers per glomerulus was evaluated in ~100 glomeruli per section using the TissueGnositics system. † *P* < 0.05 by Bonferroni’s post-hoc test compared to healthy controls, following one-way ANOVA. Images taken by Nuance system were remixed for color separation and composite images are shown. Representative images of glomeruli are shown in the bottom panel. pSmad3: DAB, Nuclei: Hematoxylin. Scale bar = 20μm. 40x objective.

**Fig 7:**
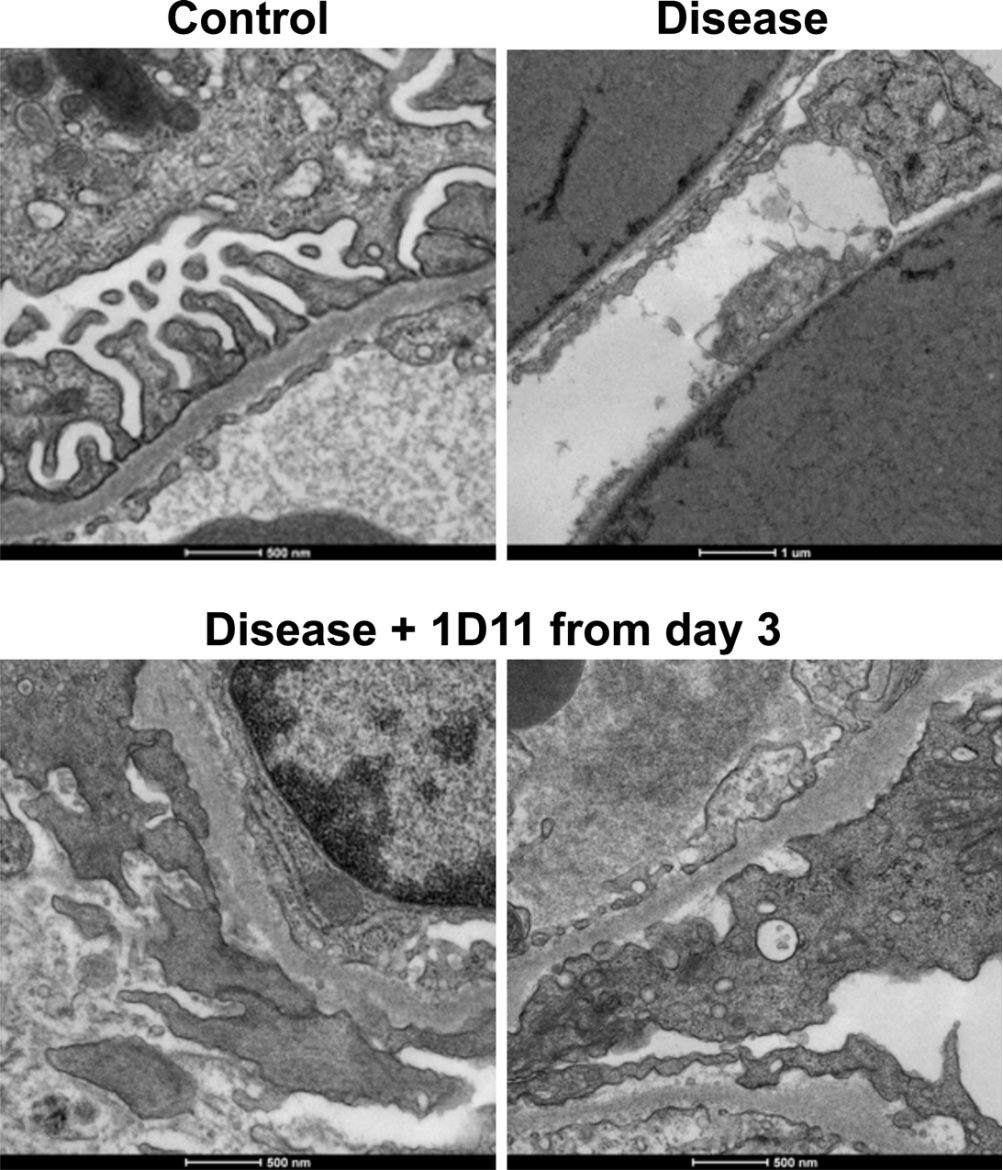
1D11 administration after the onset of proteinuria prevents podocyte detachment. Left upper panel: healthy control, right upper panel: LMB2-induced disease mouse. Lower panels: LMB2-induced disease in mice that received 1D11 after detection of proteinuria on day 3. Representative images are shown.

The degree of podocyte loss correlates with the extent of fibrotic changes, as elegantly shown by a model where diphtheria toxin kills off podocytes in a dose-dependent manner, while proteinuria could occur without overt loss of podocytes [40]. Also, in human glomerulonephritis, higher urinary podocyte excretion appears to be predictive of progression of fibrosis [41, 42]. Therefore, the apparent discrepancy in effects of 1D11 on degree of proteinuria and extent of fibrosis shown in the present study could be explained by the distinct nature of podocyte damage in the presence or absence of 1D11 treatment. As expected from the nature of the NEP25 model, podocyte number as determined by WT1-positive cells in glomeruli was significantly lower in the mice treated with LMB2 (Fig 6A). Total cell number in glomeruli and glomerular area were unchanged (Fig 6A). In comparison, podocyte numbers were relatively preserved in mice receiving 1D11 prior to LMB2 administration. 1D11 was also effective in preventing podocyte loss even when its administration was started after the onset of proteinuria (Fig 6B). Nuclear pSmad3 staining in glomeruli, as an indicator for TGF-β activity, was significantly increased in NEP25 mice receiving LMB2 and 13C4, which was reduced in mice receiving 1D11, verifying the efficacy of 1D11-mediated suppression of TGF-β signals (Fig 6C). To further evaluate these findings, selected kidney samples were examined by Transmission electron microscopy (TEM). The glomerular basement membrane (GBM) in mice that received LMB2 and control IgG (13C4) was almost completely denuded, and remaining podocytes were barely seen (Fig 7, top right). In contrast, podocytes were swollen, some forming pseudocytes, but mostly remaining attached to the GBM in mice that started receiving 1D11 3 days after the LMB2 administration and after the onset of proteinuria. Some remaining foot process structures were also observed. These findings were consistent with persisting proteinuria, but imply that podocyte detachment and/or consequent exposure of GBM might be a critical event for fibrogenesis to proceed.

## Discussion

While the fibrogenic function of TGF-β is well studied at a cellular level, TGF-β antagonism as a therapeutic approach has yet to be validated [43, 44]. In two distinct mouse models of glomerulonephritis, one that is chemically induced and the other in which podocytes are directly attacked by a chimeric toxin, mice receiving 1D11 anti-TGF-β antibody showed significantly less fibrotic changes compared to those receiving the control IgG (13C4). The treatment was effective even when the antibody was started after the onset of proteinuria. 1D11 also prevented podocyte loss, as evidenced by numbers of WT-1 positive cells and TEM evaluation. Since patients who might potentially progress to fibrotic kidney disease are generally identified only after the detection of proteinuria, the present finding emphasizes the therapeutic potential of anti-TGF-β antibody. Here, despite significant amelioration of fibrosis by 1D11, we did not observe reduction of proteinuria in either model by 1D11 treatment within the timeframe of the present experimental design. This is in agreement with our previous report where another means of TGF-β antagonism, sTβRII-Fc, does not prevent proteinuria, while fibrotic changes in ADR nephropathy were significantly reduced [20]. Similarly, genetic abrogation of TGF-β signaling by Smad3 knockout was found to be insufficient to prevent proteinuria in a streptozotocin (STZ)-induced diabetic nephropathy model, despite significant protection from glomerular hypertrophy and GBM thickening [45]. The effect of 1D11 on proteinuria has also been variably reported. In Dahl salt-sensitive hypertensive rat, 1D11 partially prevented proteinuria to a similar extent to that of anti-hypertensive medications, and the effects were additive [28]. 1D11 also prevented kidney fibrosis in Dahl rats fed a high-salt diet. Since, in that study, 1D11 also reduced mean arterial pressure in an additive manner to an angiotensin converting enzyme inhibitor (captopril), it was not certain whether 1D11 prevented podocyte injury directly or via reduction of blood pressure. 1D11 also partially reduced proteinuria in STZ-treated diabetic rats, similar to a previous report using another anti-TGF-β1 antibody [18], and the effect was additive to that of an anti-hypertensive medication (lisinopril) when treatment was started as late as 28 weeks after STZ administration [24]. In contrast, when started later (52 weeks after STZ), 1D11 ameliorated fibrosis but proteinuria persisted [25]. Artificially increased levels of TGF-β1 in the kidney, either via systemic circulation from albumin-promoter driven overexpression in liver [3], Rac1c promoter-driven expression in the juxtaglomerular apparatus [46] or hydrodynamic gene delivery to the kidney cortex [47], caused proteinuria in mice, and exposure to exogenous TGF-β induced podocyte apoptosis in vivo [48] and in vitro [49, 50], suggesting that TGF-β could damage podocytes.

TGF-β antagonism, either by 1D11 [26] or other means, such as the natural TGF-β -binding agent, decorin [15], or TβRII Fc [21, 22], ameliorates uremia and pathological changes in non-proteinuric nephritis models such as Thy1 rat nephritis that mainly involves mesangial proliferation and mesangolysis with minimal proteinuria [51], and the 5/6 nephrectomy rat model [27] that presents only a modest degree of proteinuria [52].

Our findings are consistent with our previous report that the development of proteinuria and progression of fibrosis may represent distinct processes, and that TGF-β is mainly involved in the latter. The discrepancy of our data with reports of TGF-β1 overexpression models [3, 46, 47], which demonstrate proteinuria and podocyte apoptotic changes, may be due to differences in timing and levels of TGF-β expression, or in the mode of podocyte damage. Indeed, upregulation of TGF-β is preceded by onset of proteinuria in many models [20, 53]. Further, plasma TGF-β levels reported in human patients are approximately 10–50 ng/ml [13, 54], which is much lower than that achieved in TGF-β-overexpressing mice (10–30 mg/ml) [3]. Therefore, pathogenic roles for TGF-β in the diseases might be different from that of experimental overexpression models. For example, in the NEP25 model, podocytes are directly challenged by a chimeric toxin that targets an aberrantly-expressed receptor, and therefore, the contribution of TGF-β to podocyte damage might be limited. This may also be true in many primary glomerulonephritides, in which a structural defect in podocytes due to mutation of genes encoding proteins critical for the integrity of podocyte structure may be a primary cause of the disease [55]. Together, these studies suggest that TGF-β antagonism is generally protective against glomerulosclerosis, via two facets, (1) protection from progression of fibrogenesis; and (2) protection of the podocyte, which would be more apparent when the mechanism of damage involves direct action of TGF-β on the cell.

Podocyte injury in the NEP25 model occurs in a dose-dependent manner. The highest dose of LMB2 causes massive, non-selective proteinuria and the mice die from acute renal failure and anasarca within a week, whereas low-dose LMB2 induces moderate, self-remitting proteinuria, followed by an FSGS-like lesions after 4 weeks [31]. The degree of damage also depends on levels of the toxin receptor that are expressed by the podocyte, and whether injured podocytes could propagate its damage to adjacent podocytes that do not express the toxin receptor [56]. Thus, the partial protection afforded by 1D11 treatment suggests that the initial, podocyte-toxic phase in the NEP25 model is followed by a TGF-β-dependent phase of podocytolysis. In another experimental model, in which small GTPase activity that modulates podocyte cytoskeletal structure was manipulated, proteinuria was more transient, without leading to podocyte detachment and fibrotic changes, unless there is a challenge by an additional insult [57–60]. These findings are consistent with a hypothesis in which podocyte detachment is a critical step for irreversible fibrotic changes to progress from a mere proteinuric lesion [61, 62].

TGF-β mediates multiple aspects of chronic, progressive kidney disease, affecting inflammation, apopotosis and fibrogenesis [1, 63]. Therefore, TGF-β antagonism offers layers of therapeutic potential. The present study verified the anticipated suppressive effects of 1D11 anti-TGF-β antibody on fibrotic gene activation even when administered after the onset of proteinuria in 2 distinct mouse models of glomerulonephritis. Furthermore, we showed that 1D11 also prevents podocyte detachment despite functional damage, adding another facet to the effects of 1D11 as a potential therapeutic agent to delay or prevent the progression of chronic kidney disease.

## Conclusion

1D11 anti-TGF-β antibody is a promising means to retard fibrogenesis in glomerulonephritis.
